# Genetic Adaptation of Giant Lobelias (*Lobelia aberdaric*a and *Lobelia telekii*) to Different Altitudes in East African Mountains

**DOI:** 10.3389/fpls.2016.00488

**Published:** 2016-04-12

**Authors:** Shu-Ying Zhao, Ling-Yun Chen, John K. Muchuku, Guang-Wan Hu, Qing-Feng Wang

**Affiliations:** ^1^Key Laboratory of Aquatic Botany and Watershed Ecology, Wuhan Botanical Garden, Chinese Academy of SciencesWuhan, China; ^2^Sino-Africa Joint Research Centre, Chinese Academy of SciencesWuhan, China

**Keywords:** *dN*/*dS* ratio (ω), giant lobelias, natural selection, RNA-Seq, high altitude

## Abstract

The giant lobelias in East African mountains are good models for studying molecular mechanisms of adaptation to different altitudes. In this study, we generated RNA-seq data of a middle-altitude species *Lobelia aberdarica* and a high-altitude species *L. telekii*, followed by selective pressure estimation of their orthologous genes. Our aim was to explore the important genes potentially involved in adaptation to different altitudes. About 9.3 Gb of clean nucleotides, 167,929–170,534 unigenes with total lengths of 159,762,099–171,138,936 bp for each of the two species were generated. OrthoMCL method identified 3,049 1:1 orthologous genes (each species was represented by one ortholog). Estimations of non-synonymous to synonymous rate were performed using an approximate method and a maximum likelihood method in PAML. Eighty-five orthologous genes were under positive selection. At least 8 of these genes are possibly involved in DNA repair, response to DNA damage and temperature stimulus, and regulation of gene expression, which hints on how giant lobelias adapt to high altitudinal environment that characterized by cold, low oxygen, and strong ultraviolet radiation. The negatively selected genes are over-represented in Gene Ontology terms of hydrolase, macromolecular complex assembly among others. This study sheds light on understanding the molecular mechanism of adaptation to different altitudes, and provides genomic resources for further studies of giant lobelias.

## Introduction

The upland East Africa is characterized by isolated mountains that reach alt. of 4000 m or higher. Vegetation in these mountains displays a conspicuous altitudinal zonation, starting with a montane forest belt, followed by an (subalpine) ericaceous belt, and finally an afro-alpine belt above 3500–4000 m alt. (Hedberg, 1951, 1970). The climate of montane forest is relatively temperate and seasonal, with temperatures falling below 10°C in cold season and rising to above 30°C in warm season. The belt contains moderate levels of species richness, which is higher than the surrounding lowlands (Agnew and Shirley-Agnew, 1994). Typical plants include bamboo, *Hagelia*, *Podocarpus*, etc. Species richness decreases with increase in altitude and fluctuating temperature (Hedberg, 1969). The afro-alpine belt is characterized by an extreme weather pattern with “summer every day and winter every night” [intense insolation in daytime and heavy frost at night; Hedberg (1957, 1964)]. The number of vascular plants at afro-alpine belt is significantly reduced, with only 70–150 species at each of the mountains (Hedberg, 1957). Typical plants in afro-alpine zone include the well-known giant senecios, giant lobelias among others.

**FIGURE 1:**
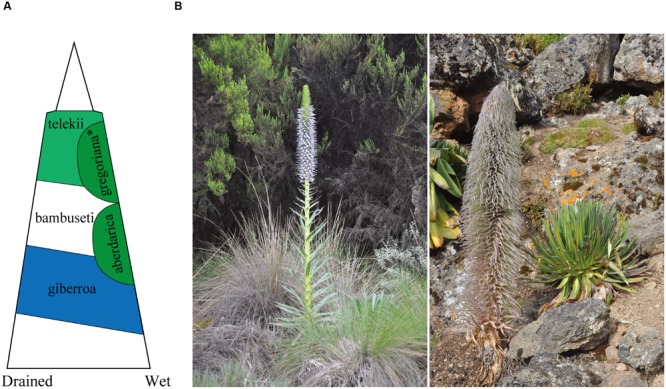
**Distribution and photos of giant lobelias. (A)** Generalized distribution on Mt Kenya along altitude and moisture [modified from Knox and Palmer (1998)]. *Lobelia gregoriana was treated as *L. deckenii* subsp. *keniensis* in Thulin (1984). **(B)** *Lobelia aberdarica* (left), photographed at Aberdare Mountains (Kenya), alt. c. 2800 m (photo: Ling-Yun Chen); *L. telekii* (right), photographed at Mt Kenya (Kenya), alt. c. 4200 m (photo: Ling-Yun Chen).

Giant lobelias (Lobeliaceae) in East African mountains are good models for studying plant adaption to different altitudes. Giant lobelias are perennial, rosette forming herbs and gradiently occur at different ecological belts of East African mountains (Thulin, 1984). The group represents an iconic example of plant adaptation to alpine conditions (Hedberg, 1964, 1969) and a conspicuous landscape of East African mountains. Five species of giant lobelias occur in Kenya and northern Tanzania (according to our observation at Mt Elgon, Cherangani hills, Aberdare mountains, Mt Kenya, Mt Meru, and Mt Kilimanjaro; **Figure 1** illustrated the general distribution of giant lobelias at Mt Kenya and photos of *L. aberdarica* and *L. telekii*). *L. telekii* Schweinf. occurs in the afro-alpine zone from alt. 3400 m to a hostile environment at high altitude (alt. 4640 m, Mt Kenya). This species is also considered to reach the highest distribution in altitude of giant lobelias in Africa. *L. aberdarica* R.E.Fr. & T.C.E.Fr. commonly occurs in moorland, high lands along streams, surrounding marshy area or mountain bogs, and montane forest edges from 2360 m to 3300 m (**Figure 1**). *L. giberroa* Hemsl. and *L. bambuseti* R.E.Fr. & T.C.E.Fr. occur in montane forest belt. *L. deckenii* (Asch.) Hemsl. occurs in the afro-alpine belt from ericaceous zone at lower altitude to lower edge of upper alpine zone (3300–4380 m). Recent studies suggested that these species are closely related, and the alpine ones were recently derived from the low altitude relatives during the Pliocene and Pleistocene (4.0–0.8 million years ago [Ma]) (Knox and Palmer, 1998; Chen et al., 2016). Chromosome number for the five species are 2*n* = 28 (Knox and Kowal, 1993).

Hedberg (1964) and Beck et al. (1982) investigated the adaptive trends in the afro-alpine flora which included *L. telekii*, *L. deckenii*, and giant senecios. These rosette plants have evolved to present a conspicuous structure good for temperature insulation. In daytime, most of their leaves unfold for photosynthesis, whereas at night they are folded up and become firmly compressed, forming a compact cabbage-like head, which maintains temperature above freezing (Hedberg, 1964). Flowers of *L. telekii* are concealed among long, hairy bracts, which can buffer vigorous daily temperature fluctuations in hostile alpine environment (Hedberg, 1964). These features were not observed in the mountain forest species such as *L. aberdarica*. The progressive adaptation of giant lobelias to afro-alpine conditions might have been facilitated by extensive volcanism through creating new habitats (Hedberg, 1970), and by induced mutations in flower buds through radiant heat shocks (Pettersson, 1961). Although previous works shed light on understanding the adaptive evolution of giant lobelias to different altitudes (Hedberg, 1951, 1957, 1964, 1969, 1970; Beck et al., 1982; Knox and Kowal, 1993; Knox and Palmer, 1998), the genes that could be involved in the adaptation remain unknown.

Acquisition of advantageous mutations by positive selection has been associated with adaptation to differentiated environments (Clark et al., 2007; Zhang et al., 2013; Poppe et al., 2015). Negative (purifying) selection plays important roles in maintaining the stability of biological structures by removal of alleles that are deleterious (Loewe, 2008). Positive and negative selection can be inferred by estimating the ratio of non-synonymous substitution rate to synonymous substitution rate (*dN*/*dS*, equivalent to ω) (Yang, 1998). Facilitated by next generation sequencing technology, the genetic basis of human and animal adaptation to different altitudes has been largely investigated by genome comparison (Yi et al., 2010) and assessing the selective pressure of orthologous genes (Simonson et al., 2010; Qiu et al., 2012; Qu et al., 2013). However, the genetic basis of plant adaptation to different altitudes has been poorly studied (but see Chapman et al., 2013; Zhang et al., 2013). Zhang et al. (2013) compared the RNA-seq data of *Primula poissonii* and *P. wilsonii*. Nevertheless, the distribution altitudes of the two species are similar^1^ (eFlora of China).

As part of a suite of works to explore the molecular mechanism of plant adaptation to high altitude, we here generated RNA-seq data for *L. aberdarica* and its closest alpine relative *L. telekii* (Knox and Palmer, 1998; Chen et al., 2016), and tested the selective pressure in orthologs of the two species. Our aims were to (1) increase the limited genetic resources of African mountain plants, and (2) identify candidate genes involved in adaptation to different altitudes by analyzing functions of the positively selected genes (PSGs) and environmental differences of the two species.

## Materials and Methods

### Materials and Sequencing

Seeds of *L. aberdarica* and *L. telekii* were collected from Aberdare mountains (00°31″27.27″ S; 36′43′17.13 E; 2925 m alt.) and Mt Kenya (00° 08′ 12. 65″ S; 037°21′16.60″ E; 4214 m alt.) respectively, in July 2014. The corresponding specimens (SAJIT-P.P1 and SAJIT-002116) were deposited at Botanische Staatssammlung München (M) and herbarium of Wuhan Botanical Garden (HIB), respectively. Tissues preserved in RNAlater storage solution did not yield high quality total RNA in our preliminary study. Therefore, seeds were grown in a plant growth incubator for 4 months with day temperature of c. 15°C and night temperature of c. 10°C. Whole plant of one individual for each species was used for total RNA extraction using RNAiso TMPlus (Takara, Qingdao, China) and then treated with RNase-free DNase I (Takara, Qingdao, China) for 45 min. Quality of RNA was checked using 2% agarose gel electrophoresis. Double stranded cDNA was sequenced using the Illumina HiSeq^TM^ 2000 sequencer (90 bp paired-end) in Beijing Genomics Institute (Wuhan, China) following the methodology in Chen et al. (2015).

### Assembling and Functional Annotation

Raw reads were cleaned by removing adaptor sequences, reads with unknown base calls (N) more than 5%, and low quality reads (>20% of the bases with a quality score≤10) using Filter_fq (an internal program of Beijing Genomics Institute). *De novo* assembly was carried out with the program Trinity v. 20130225 (Grabherr et al., 2011). Contigs were assembled to unigenes by Trinity using pair-end information. The unigenes were further processed by the TGI Clustering Tool (TGICL) v. 2.1 (Pertea et al., 2003) to remove redundancies, and assembled to acquire non-redundant unigenes as long as possible. Overlaps of at least 40 bp, and maximum length of unmatched overhangs of 20 bp were used in parameters of TGICL.

The non-redundant unigenes of the two species were annotated to NCBI non-redundant protein database (NR), Swiss-Prot protein database^2^ (Swiss-Prot), Kyoto Encyclopedia of Genes and Genomes^3^ (KEGG), Cluster of Orthologous Groups database^4^ (COG), and Gene Ontology (GO) using BLASTX (*E*-value < 10^-5^). The unigenes were also annotated to NCBI nucleotide database (NT) by BLASTN (*E*-value < 10^-5^). Sequence direction of the unigenes was determined using the best aligning results between the unigenes and the protein databases. Incongruent results from different databases were settled by a priority order of NR, Swiss-Prot, KEGG, and COG. Coding sequences (CDSs) of the unigenes were predicted by firstly aligning to NR, then Swiss-Prot, then KEGG and finally COG with BLASTX. CDSs and protein sequences were predicted by using ESTScan v. 1.1 (Iseli et al., 1999).

### Identification of Orthologous Genes

The orthologous genes between *L. aberdarica* and *L. telekii* were identified using the program OrthoMCL v.1.4 (Li et al., 2003) with all-against-all BLASTP comparisons of the predicted protein sequence. Putative orthologous relationships were identified between pairs of genomes by reciprocal best similarity pairs (Li et al., 2003). A third species can increase efficiency in computational screening for orthologs (Lee et al., 2002; Li et al., 2003; Wu et al., 2006). Therefore, protein sequences of *Vitis vinifera*, which show high similarity to the two lobelia species, were downloaded from Genoscope^5^ Redundant sequences of *V. vinifera* were removed, and used for ortholog identification. Only orthlogous clusters with a single gene for each of the three species and CDS length longer than 150 bp were kept. One strategy was further used to exclude possible paralogs: a local BLAST database was constructed using protein sequences accessed from NCBI (Auguest 2015) with the software NCBI blast+ v. 2.2.31 (Camacho et al., 2009). Protein sequences of 19 species, which showed high similarity to sequences of lobelias in our preliminary analyses, were incorporated, viz. *Amborella trichopoda*, *Arabidopsis thaliana*, *Brassica napus*, *Camelina sativa*, *Citrus sinensis*, *Cucumis sativus*, *Elaeis guineensis*, *Fragaria vesca*, *Glycine max*, *Gossypium raimondii*, *Malus domestica*, *Medicago truncatula*, *Nelumbo nucifera*, *Populus trichocarpa*, *Prunus mume*, *Ricinus communis*, *Sesamum indicum*, *V. vinifera*, and *Zea mays*. Orthologs between *L. aberdarica* and *L. telekii* were used as queries to search the local database using BLASTX with *E*-value = 10^-5^ and default settings. The matched sequence with the highest score for each gene was kept. If two orthologs within one pair were matched to different sequences, they were excluded from further analyses. After removing the sequences of *V. vinifera*, CDS of the putative orthologs between *L. aberdarica* and *L. telekii* were aligned by MUSCLE (Edgar, 2004) with default parameters. Finally, the aligned sequences were inspected in BioEdit v. 7.1.3 (Hall, 1999). All gaps and codons with mismatches were deleted. Orthologs with mismatches more than 20 bp or stop codons were excluded from further analyses.

### Estimation of Selective Pressure

Pairwise comparison was implemented to test the selective pressure of each gene. An approximate method proposed by Yang and Nielsen (2000) and a maximum likelihood (ML) method were used. The approximate analysis was conducted using yn00 in PAML toolkit v. 4.8 (Yang, 2007). The ML analysis was conducted using codeml in PAML toolkit with seqtype = 1, codonfreq = 2, runmode = -2. After preliminary analyses, orthologs with *dS* > 0.1 (Bustamante et al., 2005), or ω > 98 caused by extremely low *dS* value, were excluded to avoid potential paralogs and bias on results of selective pressure estimation.

### Functional Annotations

All orthologs were annotated to a local protein database using BLASTX in NCBI blast+ v. 2.2.31. The local database was constructed by blast+ v. 2.2.31 using all protein sequences of *Arabidopsis thaliana* accessed from NCBI (Auguest 2015). *E*-value of 1.0^-5^ and 1 BLAST hit were used. GO terms for each sequence were obtained by converting ‘GenBank Protein Accession’ to ‘GO ID’ using the website BioDBnet^6^ Orthologs were divided into three datasets: one included (PSGs, 98 > ω > 1), one included negatively selected genes (NSGs, 0 < ω < 1), and one included strongly negatively selected genes (SNSGs, ω = 0). To detect which molecular functions, biological processes and cellular component were over-represented, we compared the GO terms among the three datasets using WEGO (Ye et al., 2006). GO enrichment analysis was also conducted using agriGO (Du et al., 2010).

Only two species were used in the estimation of selective pressure. Therefore, the genes under positive selection might represent adaption to different altitudes, or other species-specific traits unrelated to altitude adaptation. In order to find out the candidates for adaptation to different altitudes, we accessed the possible functions of the positively selected genes using literature searches and gene annotations in The *Arabidopsis* Information Resource (TAIR). The positively selected genes were used as queries to search ‘TAIR10 Proteins’ dataset using BLASTX with default settings^7^ Annotations of *Arabidopsis* genes with the highest scores were used. The positively selected genes with possible functions matching the environmental differences such as cold stimulus were identified as candidates for adaptation to different altitudes.

Extremely high (≥80%) or low GC (≤25%) content have low power to accurately estimate selective pressure (Gharib and Robinson-Rechavi, 2013). GC content for each ortholog was calculated using perl scripts to check whether the value is extremely high or low. Mean GC content for the three datasets was also calculated.

## Results

### *De Novo* Assembly and Annotation of Unigenes

We generated c. 104 million clean reads, c. 9.3 Gb of nucleotides for *L. aberdarica* and *L. telekii* separately. The clean reads were submitted to the NCBI Sequence Reads Archive (nos. SRR3180742 and SRR3180743). The unigenes, which were assembled by using contigs, were 951 and 1004 bp on average with N50 of 1,951 and 1,997 bp, respectively, for the two species (Supplementary Table S1).

All the non-redundant unigenes were annotated using NR, Swiss-Prot, KEGG, COG, and NT. The results indicated that 79,825 unigenes of *L. aberdarica* (48%) and 84,296 unigenes of *L. telekii* (49%) have significant matches (*E*-value < 10^-5^). NR has the highest proportion of successful annotations, while COG has the lowest proportion. The three top-hits for the two species in the NR database were *V. vinifera*, *Lycopersicon esculentum*, and *Amygdalus persica* (Supplementary Figure S1).

### Selective Pressure Analyses

In total, 3,978 pairs of putative 1:1 orthologous genes between *L. aberdarica* and *L. telekii* were identified by OrthoMCL. After removing the pairs that were not consistent in the BLAST analyses, 3,182 pairs were retained. After removing the pairs with unexpected stop codons and mismatches more than 20 bp, 3049 pairs were retained. Sequences for the 3049 pairs were provided in Supplementary data.

**FIGURE 2:**
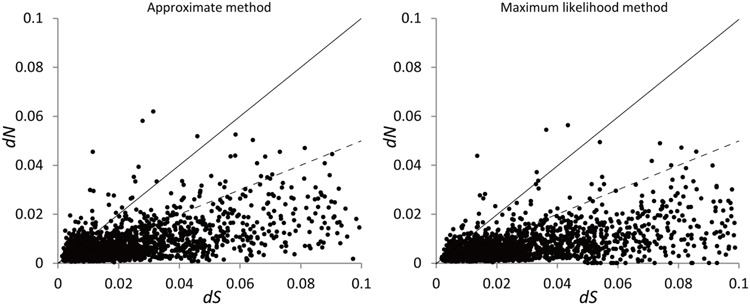
**Distribution of *dN* and *dS* for the 3,049 pairs of putative orthologous genes estimated using the approximate method with the program yn00 and maximum likelihood method with the program codeml.** The pairs with ω > 1 fall above the solid line while those with ω < 0.5 fall below the dashed line. The pairs with *dS* > 0.1 are not shown.

One hundred and sixty-eight (5.5%) pairs of orthologous genes with 98 > ω > 1 was recovered with the approximate analysis while 86 (2.8%) pairs was recovered with the ML analysis. For conciseness, one orthologous pair was counted as one gene hereafter. 85 (2.8%) pairs with 98 > ω > 1 were shared between the two analyses. 2357 (77.3%) pairs with 0 < ω < 1 were recovered in the two analyses. 480 pairs (15.7%) with ω = 0 were recovered in the two analyses. ω = 0 (caused by *dN* = 0) indicates stringent negative selection. **Table 1** summarizes the results of the two analyses; **Figure 2** plots the distribution of the values of *dN* and *dS*.

**Table 1:** Summary of the selective pressure analyses.

	1 < ω < 98	0 < ω < 1	*dN* or *dS* = 0
Approximate analysis	168	2358	480
ML analysis	86	2483	523
Co-existed between two analyses	85 (dataset 1)	2357 (dataset 2)	480 (dataset 4)
GC content (%)	46.3 (39.4–55.0)	45.7 (38.6–60.6)	46.2 (35.4–62.3)

*Numbers of orthologous pairs recovered from approximate analysis implemented in yn00 and maximum likelihood (ML) analysis implemented in codeml, numbers of pairs shared between the two analyses, and GC contents of the orthologs are shown.*

### Functional Annotations

Some of the 85 positively selected genes might be related to species-specific traits rather than altitude adaptation as only two species were used in our analyses. Annotations suggested that at least 8 of the 85 positively selected genes (PSGs) are involved in functions including DNA repair, regulation of photosynthesis, response to cold, light, or desiccation stimuli (**Table 2**). Functions of the eight genes can be associated with the environments in which giant lobelias inhabit (**Table 2**). For example, *CL8493.Contig*, which encodes a putative psbP protein, is essential for regulation and stabilization of photosystem II in higher plants (Huynh et al., 2005); Unigene78063, which encodes putative phototropic-responsive NPH3 family protein, is located in chloroplast and involved in response to light stimulus (Motchoulski and Liscum, 1999). Four of the 85 PSGs are putative zinc finger family proteins, including *Unigene36113*, *CL11459.Contig1*, *Unigene40961*, and *CL22102.Contig1*. These PSGs are multifunctional genes, involved in the regulation of transcription, nucleic acid binding among other functions. See Supplementary Table S2 for detail information of the 85 orthologous genes.

**Table 2:** Candidate genes for adaptation to different altitudes.

Seq. ID	*Arabidopsis* accession nos.	*E*-value (BLASTX)	Gene or protein names	Possible functions and biological process
*CL11124.Contig1*	AT5G43210.1	3e-39	Excinuclease ABC, C subunit, N-terminal	Nuclease activity; Involved in: DNA repair
*CL10902.Contig2*	AT4G25130.1	7e-90	A chloroplast-localized methionine sulfoxide reductase, a member of the MSRA family	Involved in: cellular protein modification process, cellular response to oxidative stress, protein repair, response to cytokinin, response to light stimulus
*CL6679.Contig*	AT5G62390.1	1e-83	A member of *Arabidopsis* BAG proteins	Involved in: apoptotic process, cellular response to cold, cellular response to heat, cellular response to unfolded protein, protein folding
*CL14161.Contig*	AT1G05260.1	1e-76	Encodes a cold-inducible cationic peroxidase that is Involved in: the stress response	Involved in: hyperosmotic salinity response, plant-type cell wall organization, response to cold, response to desiccation
*Unigene26608*	AT3G02820.1	6e-62	Zinc knuckle (CCHC-type) family protein	Involved in: cell cycle, replication fork protection, response to DNA damage stimulus
*CL8493.Contig*	AT1G77090.1	9e-87	Mog1/PsbP/DUF1795-like photosystem II reaction center PsbP family protein	Involved in: photosynthesis; Located in: chloroplast
*Unigene78063*	AT5G48130.1	3e-12	Phototropic-responsive NPH3 family protein (BTB/POZ-like)	Functions in: signal transducer activity; Involved in: response to light stimulus; Located in: chloroplast
*Unigene29384*	AT1G09540.1	2e-82	Encodes putative transcription factor	Involved in: regulation of stomatal movement, regulation of transcription, DNA-templated, response to auxin, root development

*The listed genes are a subset of the 85 positively selected genes based on annotations of TAIR, and a literature search. Since the two orthologs within an orthologous pair matched to same sequence, only sequence ID and E-values for sequences of Lobelia aberdarica are provided.*

**FIGURE 3:**
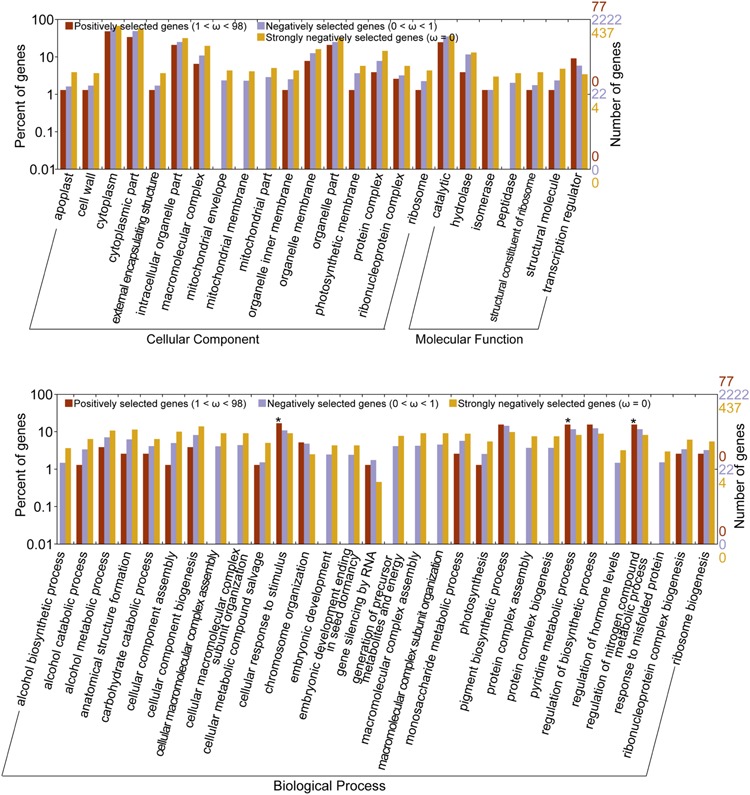
**Gene Ontology (GO) distribution plotted by WEGO.** Only the GO with significantly different level (*p*-value of Pearson Chi-Square test < 0.05) were shown. *indicates the GOs over-represented in positively selected genes. The original data for plotting this figure was provided in Supplementary data.

Gene Ontology analyses using WEGO suggested that there were significant differences (Pearson Chi-Square test, *P* < 0.05) among the three orthologous datasets at GO levels 3, 4, and 5. The negatively selected genes (0 < ω < 1) and strongly negatively selected genes (ω = 0) were over-represented in cytoplasmic part, hydrolase activity among others than the positively selected genes. The strongly negatively selected genes were over-represented in mitochondrial envelope, cellular component biogenesis among others than the negatively selected genes. The PSGs (98 > ω > 1) had a significantly higher percentage of genes with functions related to cellular response to stimulus, pyridine metabolic process, and regulation of nitrogen compound metabolic process than the strongly negatively selected genes (see Supplementary Table S3 and **Figure 3**).

Gene Ontology enrichment analysis using agriGO suggested that there was no significant enrichment (Fisher test, *P* value < 0.05) at the secondary level among the three ortholog datasets. The PSGs had a higher percentage of genes with functions related to cell part, organelle part, transcription regulator activity, and binding than the other two datasets but with *P* value > 0.05.

GC content for ortholog ranges from 35.4 to 62.3%. No orthologs have extremely high or low GC content (≥80 or≤25%). Average content for positively selected genes, negatively selected genes and strongly negatively selected genes are 46.3, 45.7, and 46.2%, respectively (**Table 1**).

## Discussion

In this study, more than 100 million of RNA-seq reads were generated and assembled into c. 160,000 unique sequences for each of *L. aberdarica* and *L. telekii*. The RNA-seq data are informative for SSR marker development and population genetic studies of the giant lobelias.

Selective pressure estimation using approximate analysis and ML analysis suggested that 168 and 86 pairs of orthologous genes were under positive selection, respectively. Taken into consideration that both methods have weaknesses [such as the ω biases when there are transition/transversion rate biases (Yang and Nielsen, 2000)], only the 85 pairs recovered in both methods were identified as PSG. *L. aberdarica* occurs in relatively mild habitats while *L. telekii* occurs in habitats with extremely strong ultraviolet radiation, fluctuating temperature and low oxygen. At least 8 of the 85 genes might be involved in adaptation to the different environments of *L. aberdarica* and *L. telekii* based on literature searches (**Table 2** and Supplementary Table S2).

*CL11124.Contig1* is a putative endonuclease subunit slx1 gene, which is required for homologous recombination and DNA repair in eukaryotic cells (Castor et al., 2013; Gaur et al., 2015). *Unigene26608* is a putative zinc knuckle family protein, which responds to DNA damage stimulus (Srivastava et al., 2010). In consideration of the DNA damage or mutation of *L. telekii* caused by heat shocks of volcanic eruptions (Pettersson, 1961), UV and frost (Hedberg, 1969; Sinha and Häder, 2002), these genes are likely important in the genetic adaptation of this species. Similarly, transcriptome analyses of *Primula poissonii* (collected at alt. 3314 m) and *P. wilsonii* (collected at alt. 2450 m) indicated that several genes related to DNA repair and homologous recombination were under positive selection and over-represented (Zhang et al., 2013). In contrast, no negatively selected genes were indicated as putative slx genes or zinc knuckle family genes, thus highlighted the roles of the two genes.

Photosynthetic capacity of *L. telekii* is sensitive to temperature change (Bodner and Beck, 1987). Photosynthesis of *L. telekii* was found to be strongly reduced when temperature above 15°C while frozen leaves regained full photosynthesis immediately after thawing (Schulze et al., 1985). *CL6679.Contig* is a putative BAG family molecular chaperone regulator seven gene (*AtBAG7*) that is an essential component of the unfolded protein response during heat and cold tolerance (Williams et al., 2010). *AtBAG7* knockouts are sensitive to heat and cold stimuli (Williams et al., 2010). *CL14161.Contig* is a putative cold inducible cationic peroxidase gene, which is in response to cold or desiccation stimulus. In this study, GO enrichment analyses also indicated that the GO terms associated with response to stimulus are over-represented in the positively selected genes (**Figure 3**). The functions of these genes are consistent with the extremely cold and sometimes dry condition that *L. telekii* faces and the peculiar mechanism of frost avoidance and freezing tolerance of the species (Hedberg, 1964; Beck et al., 1982). *CL10902.Contig2* is a putative peptide-methionine sulfoxide reductase (PMSR) gene, which is involved in cellular response to oxidative stress, oxidation-reduction process, protein repair, and response to light stimulus (Gustavsson et al., 2002; Romero et al., 2004). Zhang et al. (2013) found that several orthologs related to abiotic stress (such as oxidative stress) were under positive selection, which is consistent with our results.

Several orthologs are transcription factors involved in regulation of gene expression or DNA binding. For example, *Unigene29384* encodes a putative transcription factor, which regulates stomatal movement and transcription (**Table 2** and Supplementary Table S2). Several zinc finger proteins (ZFPs) were identified in this study. ZFPs are a large family of transcription regulators in plants for modulating several stress-responsive genes. In *Arabidopsis*, functions of C_2_H_2_ type ZFPs, including *ZAT6*, *ZAT10*, and *ZAT12*, have been well characterized. Previous studies have shown that modulation of ZFPs regulate plant responses to abiotic stresses including cold and drought (Davletova et al., 2005; Mittler et al., 2006; Shi et al., 2014). Since *L. aberdarica* and *L. telekii* occur in different altitudes of East African mountains, ZFPs might be involved in responses to temperature and humidity.

All the above genes might contribute to the adaptive evolution of the two species. However, it is possible that some of the identified PSGs are species-specific traits unrelated to habitat adaptation. On the other hand, some of the genes involved in habitat differences may remain obscure due to limited annotations in giant lobelias.

The main consequence of negative selection is the extinction of less-adapted variants (Loewe, 2008). The negatively selected genes are important for maintaining key functions of *L. aberdarica* and *L. telekii*. GO classification using WEGO suggested that the negatively selected genes were constituted of genes including functions on hydrolase, macromolecular complex assembly, and generation of precursor metabolites and energy with a higher percentage than the PSGs. These functions and processes might be involved in maintaining normal growth of the two species.

The molecular mechanisms of plant adaptation to different altitudes are complex, very few studies have explored this field. In this study, we identified 85 positively selected genes between an afro-montane forest species *L. aberdarica* and an upper afro-alpine distributed species *L. telekii*. At least 8 genes related to cold stimulus, DNA repair and regulation of gene expression were positively selected, which are consistent with characters of high altitudinal environments. This study is a tentative attempt to explore the complex molecular mechanism of plant adaptation to different altitudes using non-model plants. However, it is still limited in sampling as only two species were used, and our pairwise approach cannot distinguish in which lineage positive selection occurred. Moreover, gene turnover and expression level could also reveal environmental adaptation that is not detectable using *dN/dS* analysis. Further studies with genome sequencing and more species of African giant lobelias will be carried out to explore the adaption to different altitudes.

## Author Contributions

SYZ and QFW conceived this study; SYZ, JM, GWH, and LYC carried out experimental works, data analyses and drafted the manuscript; LYC and QFW revised the manuscript. All authors gave final approval of the version to be published.

## Conflict of Interest Statement

The authors declare that the research was conducted in the absence of any commercial or financial relationships that could be construed as a potential conflict of interest.

## Footnotes

1 http://www.efloras.org/flora_page.aspx?flora_id=2

2 http://www.expasy.ch/sprot

3 http://www.genome.jp/kegg/

4 http://www.ncbi.nlm.nih.gov/COG/

5 http://www.genoscope.cns.fr/spip/

6 http://biodbnet.abcc.ncifcrf.gov/

7 https://www.arabidopsis.org/Blast/index.jsp

## References

[B1] Agnew A. D. Q. Shirley-Agnew (1994). *Upland Kenya Wild Flowers-A Flora of the Ferns and Herbaceous Flowering Plants of Upland Kenya.* Nairobi: East Africa Natural History Society.

[B2] Beck E. Senser M. Scheibe R. Steiger H. Pongratz P. (1982). Frost avoidance and freezing tolerance in Afroalpine ‘giant rosette’plants. *Plant Cell Environ.* 5 215–222. 10.1111/1365-3040.ep11572080

[B3] Bodner M. Beck E. (1987). Effect of supercooling and freezing on photosynthesis in freezing tolerant leaves of Afroalpine ‘giant rosette’plants. *Oecologia* 72 366–371. 10.1007/BF00377565 28311131

[B4] Bustamante C. D. Fledel-Alon A. Williamson S. Nielsen R. Hubisz M. T. Glanowski S. (2005). Natural selection on protein-coding genes in the human genome. *Nature* 437 1153–1157. 10.1038/nature04240 16237444

[B5] Camacho C. Coulouris G. Avagyan V. Ma N. Papadopoulos J. Bealer K. (2009). BLAST+: architecture and applications. *BMC Bioinformatics* 10:e421 10.1186/1471-2105-10-421 PMC2803857 20003500

[B6] Castor D. Nair N. Déclais A. C. Lachaud C. Toth R. Macartney T. J. (2013). Cooperative control of holliday junction resolution and DNA repair by the SLX1 and MUS81-EME1 nucleases. *Mol. Cell* 52 221–233. 10.1016/j.molcel.2013.08.036 24076219 PMC3808987

[B7] Chapman M. A. Hiscock S. J. Filatov D. A. (2013). Genomic divergence during speciation driven by adaptation to altitude. *Mol. Biol. Evol.* 30 2553–2567. 10.1093/molbev/mst168 24077768 PMC3840311

[B8] Chen L. Y. Renner S. S. Wang Q. F. (2016). East Asian *Lobelioideae* and ancient divergence of a giant rosette *Lobelia* in Himalayan Bhutan. *Taxon.* 1 10.12705/652.6

[B9] Chen L. Y. Zhao S. Y. Wang Q. F. Moody M. L. (2015). Transcriptome sequencing of three *Ranunculus* species (*Ranunculaceae*) reveals candidate genes in adaptation from terrestrial to aquatic habitats. *Sci. Rep.* 5 e10098. 10.1038/srep10098 PMC4438715 25993393

[B10] Clark A. G. Eisen M. B. Smith D. R. Bergman C. M. Oliver B. Markow T. A. (2007). Evolution of genes and genomes on the *Drosophila* phylogeny. *Nature* 450 203–218. 10.1038/nature06341 17994087

[B11] Davletova S. Schlauch K. Coutu J. Mittler R. (2005). The zinc-finger protein Zat12 plays a central role in reactive oxygen and abiotic stress signaling in *Arabidopsis*. *Plant Physiol.* 139 847–856. 10.1104/pp.105.068254 16183833 PMC1256000

[B12] Du Z. Zhou X. Ling Y. Zhang Z. Su Z. (2010). agriGO: a GO analysis toolkit for the agricultural community. *Nucleic. Acids Res.* 38 1–10. 10.1093/nar/gkq310 20435677 PMC2896167

[B13] Edgar R. C. (2004). MUSCLE: multiple sequence alignment with high accuracy and high throughput. *Nucleic. Acids Res.* 32 1792–1797. 10.1093/nar/gkh340 15034147 PMC390337

[B14] Gaur V. Wyatt H. D. Komorowska W. Szczepanowski R. H. De Sanctis D. Gorecka K. M. (2015). Structural and mechanistic analysis of the Slx1-Slx4 endonuclease. *Cell Rep.* 10 1467–1476. 10.1016/j.celrep.2015.02.019 PMC4407285 25753413

[B15] Gharib W. H. Robinson-Rechavi M. (2013). The branch-site test of positive selection is surprisingly robust but lacks power under synonymous substitution saturation and variation in GC. *Mol. Biol. Evol.* 30 1675–1686. 10.1093/molbev/mst062 23558341 PMC3684852

[B16] Grabherr M. G. Haas B. J. Yassour M. Levin J. Z. Thompson D. A. Amit I. (2011). Full-length transcriptome assembly from RNA-Seq data without a reference genome. *Nat. Biotechnol.* 29 644–652. 10.1038/nbt.1883 21572440 PMC3571712

[B17] Gustavsson N. Kokke B. P. Härndahl U. Silow M. Bechtold U. Poghosyan Z. (2002). A peptide methionine sulfoxide reductase highly expressed in photosynthetic tissue in *Arabidopsis thaliana* can protect the chaperone-like activity of a chloroplast-localized small heat shock protein. *Plant J.* 29 545–553. 10.1046/j.1365-313X.2002.029005545.x 11874568

[B18] Hall T. A. (1999). BioEdit: a user-friendly biological sequence alignment editor and analysis program for Windows 95/98/NT. *Nucleic Acids Symp. Ser.* 41 95–98.

[B19] Hedberg O. (1951). Vegetation belts of the East African mountains. *Svensk. Bot. Tidsk* 45 140–202.

[B20] Hedberg O. (1957). *Afroalpine Vascular Plants: A Taxonomic Revision.* Uppsala: A. B. Lundequistska Bokhandeln.

[B21] Hedberg O. (1964). Features of afroalpine plant ecology. *Acta Phytogeogr. Suec.* 49 1–144.

[B22] Hedberg O. (1969). Evolution and speciation in a tropical high mountain flora. *Biol. J. Linn. Soc. Lond.* 1 135–148. 10.1111/j.1095-8312.1969.tb01816.x

[B23] Hedberg O. (1970). Evolution of the afroalpine flora. *Biotropica* 2 16–23. 10.2307/2989783

[B24] Huynh L. N. Vantoai T. Streeter J. Banowetz G. (2005). Regulation of flooding tolerance of *SAG12:ipt Arabidopsis* plants by cytokinin. *J. Exp. Bot.* 56 1397–1407. 10.1093/jxb/eri141 15797940

[B25] Iseli C. Jongeneel C. V. Bucher P. (1999). ESTScan: a program for detecting, evaluating, and reconstructing potential coding regions in EST sequences. *Proc. Int. Conf. Intell. Syst. Mol. Biol.* 1999 138–148. 10786296

[B26] Knox E. B. Kowal R. R. (1993). Chromosome numbers of the East African giant senecios and giant lobelias and their evolutionary significance. *Am. J. Bot.* 847–853. 10.2307/2445604

[B27] Knox E. B. Palmer J. D. (1998). Chloroplast DNA evidence on the origin and radiation of the giant lobelias in eastern Africa. *Syst. Bot.* 23 109–149. 10.2307/2419583

[B28] Lee Y. Sultana R. Pertea G. Cho J. Karamycheva S. Tsai J. (2002). Cross-referencing eukaryotic genomes: TIGR orthologous gene alignments (TOGA). *Genome Res.* 12 493–502. 10.1101/gr.212002 11875039 PMC155294

[B29] Li L. Stoeckert C. J. Jr. Roos D. S. (2003). OrthoMCL: identification of ortholog groups for eukaryotic genomes. *Genome Res.* 13 2178–2189. 10.1101/gr.1224503 12952885 PMC403725

[B30] Loewe L. (2008). Negative selection. *Nat. Education* 1 59.

[B31] Mittler R. Kim Y. Song L. Coutu J. Coutu A. Ciftci-Yilmaz S. (2006). Gain-and loss-of-function mutations in Zat10 enhance the tolerance of plants to abiotic stress. *FEBS Lett.* 580 6537–6542. 10.1016/j.febslet.2006.11.002 17112521 PMC1773020

[B32] Motchoulski A. Liscum E. (1999). *Arabidopsis* NPH3: a NPH1 photoreceptor-interacting protein essential for phototropism. *Science* 286 961–964. 10.1126/science.286.5441.961 10542152

[B33] Pertea G. Huang X. Liang F. Antonescu V. Sultana R. Karamycheva S. (2003). TIGR Gene Indices clustering tools (TGICL): a software system for fast clustering of large EST datasets. *Bioinformatics* 19 651–652. 10.1093/bioinformatics/btg034 12651724

[B34] Pettersson B. (1961). Mutagenic effect of radiant heat shocks on phanerogamous plants. *Nature* 191 1167–1169. 10.1038/1911167a0

[B35] Poppe S. Dorsheimer L. Happel P. Stukenbrock E. H. (2015). Rapidly evolving genes are key players in host specialization and virulence of the fungal wheat pathogen *Zymoseptoria tritici* (*Mycosphaerella graminicola*). *PLoS Pathog.* 11:e1005055 10.1371/journal.ppat.1005055 PMC4520584 26225424

[B36] Qiu Q. Zhang G. Ma T. Qian W. Wang J. Ye Z. (2012). The yak genome and adaptation to life at high altitude. *Nat. Genet.* 44 946–949. 10.1038/ng.2343 22751099

[B37] Qu Y. Zhao H. Han N. Zhou G. Song G. Gao B. (2013). Ground tit genome reveals avian adaptation to living at high altitudes in the *Tibetan plateau*. *Nat. Commun.* 4:e2071 10.1038/ncomms3071 23817352

[B38] Romero H. M. Berlett B. S. Jensen P. J. Pell E. J. Tien M. (2004). Investigations into the role of the plastidial peptide methionine sulfoxide reductase in response to oxidative stress in *Arabidopsis*. *Plant Physiol.* 136 3784–3794. 10.1104/pp.104.046656 15516509 PMC527175

[B39] Schulze E. -D. Beck E. Scheibe R. Ziegler P. (1985). Carbon dioxide assimilation and stomatal response of afroalpine giant rosette plants. *Oecologia* 65 207–213. 10.1007/BF00379219 28310667

[B40] Shi H. Wang X. Ye T. Chen F. Deng J. Yang P. (2014). The Cysteine2/Histidine2-Type transcription factor ZINC FINGER OF *ARABIDOPSIS* THALIANA6 modulates biotic and abiotic stress responses by activating salicylic acid-related genes and C-REPEAT-BINDING FACTOR genes in *Arabidopsis*. *Plant Physiol.* 165 1367–1379. 10.1104/pp.114.242404 24834923 PMC4081343

[B41] Simonson T. S. Yang Y. Huff C. D. Yun H. Qin G. Witherspoon D. J. (2010). Genetic evidence for high-altitude adaptation in Tibet. *Science* 329 72–75. 10.1126/science.1189406 20466884

[B42] Sinha R. P. Häder D. -P. (2002). UV-induced DNA damage and repair: a review. *Photochem. Photobiol. Sci.* 1 225–236. 10.1039/b201230h 12661961

[B43] Srivastava G. P. Li P. Liu J. Xu D. (2010). Identification of transcription factor’s targets using tissue-specific transcriptomic data in *Arabidopsis thaliana*. *BMC Syst. Biol.* 4:S2 10.1186/1752-0509-4-S2-S2 PMC2982689 20840729

[B44] Thulin M. (1984). “Lobeliaceae,” in *Flora of Tropical East Africa*, ed. Polhill R. M. (Rotterdam: Balkema), 1–59.

[B45] Williams B. Kabbage M. Britt R. Dickman M. B. (2010). AtBAG7, an *Arabidopsis* Bcl-2–associated athanogene, resides in the endoplasmic reticulum and is involved in the unfolded protein response. *Proc. Natl. Acad. Sci. U.S.A.* 107 6088–6093. 10.1073/pnas.0912670107 20231441 PMC2851922

[B46] Wu F. Mueller L. A. Crouzillat D. Pétiard V. Tanksley S. D. (2006). Combining bioinformatics and phylogenetics to identify large sets of single-copy orthologous genes (COSII) for comparative, evolutionary and systematic studies: a test case in the euasterid plant clade. *Genetics* 174 1407–1420. 10.1534/genetics.106.062455 16951058 PMC1667096

[B47] Yang Z. (1998). Likelihood ratio tests for detecting positive selection and application to primate lysozyme evolution. *Mol. Biol. Evol.* 15 568–573. 10.1093/oxfordjournals.molbev.a025957 9580986

[B48] Yang Z. (2007). PAML 4: phylogenetic analysis by maximum likelihood. *Mol. Biol. Evol.* 24 1586–1591. 10.1093/molbev/msm088 17483113

[B49] Yang Z. Nielsen R. (2000). Estimating synonymous and nonsynonymous substitution rates under realistic evolutionary models. *Mol. Biol. Evol.* 17 32–43. 10.1093/oxfordjournals.molbev.a026236 10666704

[B50] Ye J. Fang L. Zheng H. Zhang Y. Chen J. Zhang Z. (2006). WEGO: a web tool for plotting GO annotations. *Nucleic. Acids Res.* 34 W293–W297. 10.1093/nar/gkl031 16845012 PMC1538768

[B51] Yi X. Liang Y. Huerta-Sanchez E. Jin X. Cuo Z. X. P. Pool J. E. (2010). Sequencing of 50 human exomes reveals adaptation to high altitude. *Science* 329 75–78. 10.1126/science.1190371 20595611 PMC3711608

[B52] Zhang L. Yan H. F. Wu W. Yu H. Ge X. J. (2013). Comparative transcriptome analysis and marker development of two closely related Primrose species (*Primula poissonii* and *Primula wilsonii*). *BMC Genomics* 14:e329 10.1186/1471-2164-14-329 PMC3658987 23672467

