# Supplementary material for: Genetic Adaptation of Giant Lobelias (Lobelia aberdarica and Lobelia telekii) to Different Altitudes in East African Mountains
Source: Front Plant Sci. 2016 Apr 12;7:488. doi: 10.3389/fpls.2016.00488 (PMC4828460; doi:10.3389/fpls.2016.00488)
Supplement: Supplementary file 1 [file Table_1.DOCX]

**Supplementary Table 1**. *De novo* assembly from transcriptomes of *Lobelia aberdarica* and *L. telekii*, and annotations with E-value <10^-5^.

| **Sequences** | ***L. aberdarica*** | ***L. telekii*** |
| --- | --- | --- |
| **Clean reads** |  |  |
| No. of clean reads | 104,240,858 | 103,144,108 |
| No. of nucleotides (nt) | 9,381,677,220 | 9,282,969,720 |
| Q20 percentage | 98.41% | 98.40% |
| N percentage | 0.00% | 0.00% |
| GC percentage | 45.05% | 44.40% |
| **Contigs** |  |  |
| No. of contigs | 265,484 | 260,007 |
| Total length (nt) | 84,958,510 | 84,484,651 |
| Mean length (nt) | 320 | 325 |
| N50 | 549 | 576 |
| **Unigenes** |  |  |
| No. of unigenes | 167,929 | 170,534 |
| Total length (nt) | 159,762,099 | 171,138,936 |
| Mean length (nt) | 951 | 1004 |
| N50 | 1951 | 1997 |
| **Annotated unigenes number and percentage** | | |
| NR | 76,034 (45%) | 80,600 (47%) |
| Swiss-prot | 48,221 (29%) | 51,146 (30%) |
| KEGG | 45,038 (27%) | 47,769 (28%) |
| COG | 30,919 (18%) | 33,006 (19%) |
| GO | 53,267 (32%) | 61,971 (36%) |
| NT | 65,498 (39%) | 69,986 (41%) |
| Total | 79,825 (48%) | 84,296 (49%) |
