# Supplementary material for: Genetic Adaptation of Giant Lobelias (Lobelia aberdarica and Lobelia telekii) to Different Altitudes in East African Mountains
Source: Front Plant Sci. 2016 Apr 12;7:488. doi: 10.3389/fpls.2016.00488 (PMC4828460; doi:10.3389/fpls.2016.00488)
Supplement: Supplementary file 2 [file Table_2.DOCX]

**Supplementary Table 2**. Annotation of the positively selected genes between *Lobelia aberdaric*a and *L. telekii*. Orthologs were annotated to NR database and TAIR 10 protein database. Since in most cases the two genes within one orthologous pair were matched to same sequence, only E values for the *L. aberdarica* sequences are provided. 'Na' stands for not available of information.

| **Seq. ID of *L. aberdarica*** | **Seq. ID of *L. telekii*** | **NR. Accession nos.** | **NR database description** | **Arabidopsis accession nos. (TAIR)** | **Gene or protein names (TAIR)** | **Possible functions and biological process (TAIR**) |
| --- | --- | --- | --- | --- | --- | --- |
| CL4437.Contig1 | CL2341.Contig1 | XP_009786317.1 0.0 | PREDICTED: G-type lectin S-receptor-like serine/threonine-protein kinase SD2-5 [Nicotiana sylvestris] | AT4G32300.1 0.0 | Unkown protein | Functions in: carbohydrate binding, protein kinase activity, kinase activity; Involved in: protein amino acid autophosphorylation; Located in: plasma membrane |
| Unigene743 | CL11009.Contig2 | XP_002273643.2 0.0 | PREDICTED: folic acid synthesis protein fol1 [Vitis vinifera] | AT4G30000.1 0.0 | Dihydropterin pyrophosphokinase / Dihydropteroate synthase | Functions in: 2-amino-4-hydroxy-6-hydroxymethyldihydropteridine diphosphokinase activity, dihydropteroate synthase activity; Involved in: pteridine and derivative metabolic process, cellular metabolic process, folic acid and derivative biosynthetic process |
| CL269.Contig4 | Unigene38592 | XP_012089338.1 2e-142 | PREDICTED: uncharacterized protein LOC105647746 isoform X1 [Jatropha curcas] | AT1G10095.1 3e-87 | Protein prenylyltransferase superfamily protein | Functions in: protein prenyltransferase activity; Involved in: protein amino acid prenylation; Located in: cellular_component unknown |
| Unigene28459 | CL3331.Contig2 | XP_002278445.1 5e-45 | PREDICTED: uncharacterized protein LOC100266209 [Vitis vinifera] | AT1G69610.1 2e-16 | Unkown protein | Functions in: structural constituent of ribosome; Involved in: N-terminal protein myristoylation, translation; Located in: ribosome, intracellular |
| Unigene32497 | CL3436.Contig6 | XP_009587265.1 0.0 | PREDICTED: protein MEMO1 [Nicotiana tomentosiformis] | AT2G25280.1 e-137 | Unkown protein | UPF0103/Mediator of ErbB2-driven cell motility |
| CL4173.Contig3 | Unigene24134 | CBI35828.3 2e-77 | unnamed protein product [Vitis vinifera] | AT3G14170.1 1e-41 | Unkown protein | Plant protein of unknown function (DUF936) |
| CL4386.Contig1 | CL9981.Contig1 | XP_002281432.1 0.0 | PREDICTED: probable LRR receptor-like serine/threonine-protein kinase At1g67720 [Vitis vinifera] | AT3G05990.1 e-180 | Leucine-rich repeat (LRR) family protein | Involved in: signal transduction; Located in: endomembrane system |
| CL8332.Contig4 | CL15482.Contig2 | CDP02004.1 1e-173 | unnamed protein product [Coffea canephora] | AT5G66530.2 e-125 | Galactose mutarotase-like superfamily protein | Functions in: carbohydrate binding, isomerase activity, aldose 1-epimerase activity, catalytic activity; Involved in: galactose metabolic process, carbohydrate metabolic process; Located in: apoplast, chloroplast, chloroplast stroma |
| CL22404.Contig2 | CL14798.Contig1 | XP_011089542.1 0.0 | PREDICTED: LOW QUALITY PROTEIN: uncharacterized protein LOC105170443 [Sesamum indicum] | AT5G25590.1 e-179 | Unkown protein | Involved in: N-terminal protein myristoylation; EXPRESSED IN: 20 plant structures; EXPRESSED DURING: 13 growth stages; CONTAINS InterPro DOMAIN/s: Protein of unknown function DUF630 |
| Unigene31510 | CL5436.Contig1 | XP_012082424.1 0.0 | PREDICTED: histidine--tRNA ligase, cytoplasmic [Jatropha curcas] | AT3G46100.1 0.0 | histidyl-tRNA synthetase | Involved in: histidyl-tRNA aminoacylation; Located in: chloroplast, mitochondrion; has ATP binding, histidine-tRNA ligase activity |
| CL5814.Contig1 | Unigene2017 | CDP05977.1 0.0 | unnamed protein product [Coffea canephora] | AT1G53210.1 6e-63 | ATNCL, NA+/CA2+ EXCHANGER, NCL | Encodes a Na+/Ca 2+ exchanger-like protein that participates in the maintenance of Ca 2+ homeostasis; Involved in: calcium ion homeostasis, calcium ion transmembrane transport, cellular response to salt stress, sodium ion transmembrane transport; Functions in calcium ion binding; has calcium:sodium antiporter activity |
| CL11124.Contig1 | CL5384.Contig2 | XP_008231532.1 2e-55 | PREDICTED: structure-specific endonuclease subunit slx1 [Prunus mume] | AT5G43210.1 3e-39 | Excinuclease ABC, C subunit, N-terminal | Functions in: nuclease activity; Involved in: DNA repair; Located in: intracellular; CONTAINS InterPro DOMAIN/s: Excinuclease ABC, C subunit, N-terminal (InterPro:IPR000305) |
| CL649.Contig1 | Unigene26547 | XP_007030683.1 9e-24 | Uncharacterized protein TCM_026440 [Theobroma cacao] | na | Na | Na |
| CL6679.Contig1 | Unigene35181 | XP_009796142.1 6e-142 | PREDICTED: BAG family molecular chaperone regulator 7 [Nicotiana sylvestris] | AT5G62390.1 1e-83 | A member of Arabidopsis BAG (AtBAG7, Bcl-2-associated athanogene) proteins | Involved in: apoptotic process, cellular response to cold, cellular response to heat, cellular response to unfolded protein, protein folding; Located in: chloroplast, endoplasmic reticulum, plasma membrane, plasmodesma; Functions in calmodulin binding, protein binding; has calmodulin binding |
| Unigene3220 | CL6823.Contig4 | XP_009792802.1 2e-45 | PREDICTED: uncharacterized protein LOC104239780 [Nicotiana sylvestris] | AT2G17580.1 3e-31 | Polynucleotide adenylyltransferase family protein | Functions in: RNA binding, nucleotidyltransferase activity; Involved in: RNA processing; Located in: chloroplast |
| CL8099.Contig2 | Unigene38219 | XP_010661405.1 2e-67 | PREDICTED: plasminogen activator inhibitor 1 RNA-binding protein [Vitis vinifera] | AT4G16830.1 8e-40 | Encodes a perinuclear and cytoplasmically localized mRNA binding protein； | Involved in: cellular response to osmotic stress, cellular response to salt stress; expressed in: perinuclear region of cytoplasm; Located in: cytoplasm, nucleus; Functions in RNA binding, mRNA binding |
| CL8178.Contig1 | CL9819.Contig6 | XP_002285842.1 3e-41 | PREDICTED: uncharacterized protein LOC100262661 [Vitis vinifera] | AT4G08240.1 4e-33 | Unkown protein | Function unknown |
| CL9668.Contig1 | CL9480.Contig1 | XP_007047483.1 0.0 | SCL domain class transcription factor [Theobroma cacao] | AT3G49950.1 e-147 | GRAS family transcription factor | Involved in: regulation of transcription, DNA-templated, transcription, DNA-templated; Involved in: regulation of transcription, DNA-templated, transcription, DNA-templated; has transcription factor activity, sequence-specific DNA binding |
| CL291.Contig1 | CL15631.Contig1 | ABE11612.1 6e-54 | SWIb domain-containing protein [Solanum chacoense] | AT4G26810.2 4e-41 | SWIB/MDM2 domain superfamily protein | Function unknown |
| Unigene2610 | Unigene3979 | CDP18098.1 3e-144 | unnamed protein product [Coffea canephora] | AT3G05330.1 3e-67 | Encodes a protein with moderate sequence similarity to the maize microtubule-binding protein TANGLED1 | Involved in: phragmoplast assembly; Located in: microtubule associated complex, mitochondrion, nucleolus, phragmoplast, preprophase band |
| Unigene39114 | Unigene39261 | CDP11871.1 3e-124 | unnamed protein product [Coffea canephora] | AT4G03260.2 3e-82 | Outer arm dynein light chain 1 protein | Involved in: protein autophosphorylation; Located in: nucleus; has protein serine/threonine kinase activity |
| Unigene19168 | Unigene883 | XP_006368201.1 1e-11 | hypothetical protein POPTR_0001s00460g [Populus trichocarpa] | AT4G16515.1 7e-05 | Encodes a root meristem growth factor | Involved in: cell differentiation, lateral root development, positive gravitropism, positive regulation of cell proliferation, regulation of auxin polar transport, regulation of protein localization, root system development; Located in: extracellular region, extracellular space; has growth factor activity, molecular function |
| CL15653.Contig2 | CL1568.Contig2 | XP_011098464.1 1e-149 | PREDICTED: cinnamoyl-CoA reductase 2 [Sesamum indicum] | AT5G14700.1 8e-91 | NAD(P)-binding Rossmann-fold superfamily protein | Functions in: coenzyme binding, binding, cinnamoyl-CoA reductase activity, catalytic activity; Involved in: lignin biosynthetic process, cellular metabolic process, metabolic process; has catalytic activity, cinnamoyl-CoA reductase activity, coenzyme binding |
| Unigene45240 | Unigene45539 | XP_008385426.1 2e-93 | PREDICTED: heme-binding protein 2-like [Malus domestica] | AT1G17100.1 4e-34 | SOUL heme-binding family protein | Functions in: binding; Involved in: biological process unknown; Located in: plasma membrane, vacuole |
| Unigene36113 | Unigene35708 | XP_002284912.1 3e-103 | PREDICTED: uncharacterized protein LOC100250124 [Vitis vinifera] | AT5G62460.1 1e-62 | RING/FYVE/PHD zinc finger superfamily protein | Functions in: zinc ion binding |
| Unigene29542 | Unigene16114 | XP_002284912.1 5e-103 | PREDICTED: uncharacterized protein LOC100250124 [Vitis vinifera] | AT1G74780.1 e-172 | Nodulin-lik/Major Facilitator Superfamily protein | Involved in: transmembrane transport; Located in: endomembrane system |
| Unigene35250 | Unigene1577 | XP_007018498.1 2e-126 | Galactose oxidase/kelch repeat superfamily protein [Theobroma cacao] | AT5G43190.1 5e-80 | Galactose oxidase/kelch repeat superfamily protein | Involved in: ubiquitin-dependent protein catabolic process; has ubiquitin-protein transferase activity |
| Unigene9210 | Unigene46438 | XP_007017424.1 3e-16 | Uncharacterized protein TCM_033960 [Theobroma cacao] | Na | Na | Na |
| Unigene4026 | Unigene2015 | XP_002267616.1 0.0 | PREDICTED: two-component response regulator ARR11 [Vitis vinifera] | AT1G67710.1 e-105 | Encodes an Arabidopsis response regulator (ARR) protein that acts in concert with other type-B ARRs in the cytokinin signaling pathway | Involved in: cytokinin-activated signaling pathway, phosphorelay signal transduction system, regulation of root meristem growth, regulation of transcription, DNA-templated, response to cytokinin, transcription, DNA-templated; Located in: nucleus; has DNA binding, chromatin binding, phosphorelay response regulator activity, protein binding, transcription factor activity, sequence-specific DNA binding |
| Unigene50198 | Unigene17398 | KHN13237.1 5e-86 | Nuclear distribution protein PAC1 [Glycine soja] | AT4G25440.1 8e-05 | zinc finger WD40 repeat protein 1 (ZFWD1) | Functions in: zinc ion binding, nucleic acid binding; Involved in: biological process unknown; Located in: cellular component unknown |
| CL11459.Contig1 | CL17051.Contig1 | XP_002284740.2 4e-83 | PREDICTED: uncharacterized protein LOC100254082 [Vitis vinifera] | AT1G76010.1 2e-48 | Alba DNA/RNA-binding protein | Functions in: nucleic acid binding; Involved in: biological process unknown; Located in: cellular component unknown |
| Unigene16993 | CL15769.Contig1 | XP_006356376.1 4e-98 | PREDICTED: cucumber peeling cupredoxin-like [Solanum tuberosum] | AT1G08500.1 4e-63 | early nodulin-like protein 18 (ENODL18) | Functions in: electron carrier activity, copper ion binding; Located in: anchored to membrane |
| CL15777.Contig1 | Unigene2639 | XP_006349409.1 1e-83 | PREDICTED: partner of Y14 and mago-like [Solanum tuberosum] | AT1G11400.3(1.2) 5e-49 | The PYM gene encodes a protein capable of interacting with MAGO, and Y14, whose orthologs form part of the exon junction complex in animal cells | Involved in: positive regulation of gene expression; Located in: cytoplasm, nucleolus, nucleoplasm, nucleus; Functions in protein binding |
| Unigene17059 | Unigene34377 | XP_009766201.1 2e-103 | PREDICTED: FRIGIDA-like protein 4a [Nicotiana sylvestris] | AT3G22440.1 5e-32 | FRIGIDA-like protein | Involved in: cell differentiation, flower development; Located in: nucleus; has molecular function |
| Unigene45942 | Unigene28107 | XP_002316247.1 0.0 | UDP-glucoronosyl/UDP-glucosyl transferase family protein [Populus trichocarpa] | AT3G11340 e-111 | Encodes a glucosyltransferase that conjugates isoleucic acid and modulates plant defense and senescence | Involved in: defense response, defense response to fungus, leaf senescence; Located in: nucleus; has UDP-glycosyltransferase activity, glucosyltransferase activity |
| Unigene20936 | Unigene19612 | XP_004234947.1 6e-93 | PREDICTED: uncharacterized protein LOC101252159 [Solanum lycopersicum] | AT5G62280.1 5E-57 | Protein of unknown function (DUF1442) | Function unknown |
| Unigene16883 | Unigene45824 | XP_008218419.1 3e-34 | PREDICTED: myb-like protein X [Prunus mume] | AT1G65090.3 1E-15 | Unknown protein | Located in: cytoplasm, integral component of membrane, nucleus |
| CL3994.Contig1 | CL1157.Contig2 | XP_009777574.1 2e-50 | PREDICTED: uncharacterized protein LOC104227115 isoform X1 [Nicotiana sylvestris] | AT5G62550.1 2E-10 | Unknown protein | Located in: plasma membrane |
| Unigene45302 | Unigene45627 | CDP09783.1 9e-70 | unnamed protein product [Coffea canephora] | AT1G63220.1 1E-53 | Calcium-dependent lipid-binding (CaLB domain) family protein | Located in: cytoplasm |
| Unigene30032 | Unigene16474 | XP_006437179.1 2e-73 | hypothetical protein CICLE_v10033364mg [Citrus clementina] | AT2G42900.1 1E-38 | Plant basic secretory protein (BSP) family protein | |
| Unigene27017 | Unigene19649 | XP_003633019.2 1e-48 | PREDICTED: uncharacterized protein LOC100853536 [Vitis vinifera] | AT1G28410.1 4E-23 | Unknown protein | Located in: endomembrane system |
| CL11595.Contig3 | CL8069.Contig2 | XP_002273844.1 0.0 | PREDICTED: UPF0187 protein At3g61320, chloroplastic [Vitis vinifera] | AT3G61320.1 1E-129 | Bestrophin-like protein | Located in: chloroplast, chloroplast membrane, integral component of membrane |
| Unigene35784 | Unigene33427 | AAP86602.1 2e-112 | eukaryotic translation initiation factor 4E [Lactuca sativa] | AT4G18040.1 7E-82 | eIF4E protein. The cum1 mutation affects the local spreading of CMV within the inoculated leaf, delaying accumulation of cucumber mosaic virus coat protein | Involved in: regulation of translation, response to virus, translational initiation; Located in: cytoplasm, cytoplasmic mRNA processing body, cytoplasmic stress granule, nucleolus, nucleus; Functions in RNA binding, RNA cap binding, protein binding; has translation initiation factor activity |
| Unigene43924 | Unigene16555 | XP_007029984.1 2e-43 | Uncharacterized protein TCM_025839 [Theobroma cacao] | AT5G37480.1 1E-22 | Unknown protein | Located in: integral component of membrane |
| Unigene20376 | Unigene17908 | XP_011089348.1 9e-153 | PREDICTED: exonuclease V, chloroplastic-like [Sesamum indicum] | AT5G60370.1 3E-85 | Unknown protein | has 4 iron, 4 sulfur cluster binding, DNA binding, metal ion binding, single-stranded DNA 5'-3' exodeoxyribonuclease activity |
| Unigene45469 | Unigene16475 | XP_002275895.1 2e-78 | PREDICTED: LDLR chaperone MESD [Vitis vinifera] | AT2G46000.1 2E-45 | Unknown protein | Located in: endomembrane system |
| Unigene29384 | CL16066.Contig1 | XP_011087570.1 9e-140 | PREDICTED: transcription factor MYB86-like [Sesamum indicum] | AT1G09540.1 2E-82 | Encodes putative transcription factor. Mutants lack of mucilage extrusion from the seeds during imbibition. Reduced quantities of mucilage are deposited during the development of the seed coat epidermis in myb61 mutants | Involved in: regulation of stomatal movement, regulation of transcription, DNA-templated, response to auxin, root development, seed coat development, vasculature development, xylem development; Located in: nucleus; Functions in DNA binding; has DNA binding, chromatin binding, transcription factor activity, sequence-specific DNA binding |
| Unigene78063 | Unigene28378 | XP_009786672.1 2e-38 | BTB/POZ domain-containing protein | AT5G48130.1 3E-12 | Phototropic-responsive NPH3 family protein (BTB/POZ-like) | Functions in: signal transducer activity; Involved in: response to light stimulus; Located in: chloroplast |
| CL16506.Contig1 | CL11698.Contig3 | XP_011036546.1 0.0 | PREDICTED: pectinesterase 3-like [Populus euphratica] | AT1G53840.1 0 | encodes a pectin methylesterase | Involved in: cell wall modification, pectin catabolic process; Located in: Golgi apparatus, endosome, extracellular region, membrane, nucleus, plant-type cell wall, plasma membrane, plasmodesma, trans-Golgi network, vacuolar membrane; has pectinesterase activity, pectinesterase inhibitor activity, protein binding |
| Unigene12282 | Unigene17716 | XP_011082093.1 8e-78 | PREDICTED: putative RNA methyltransferase At5g10620 [Sesamum indicum] | AT5G10620.1 6E-53 | methyltransferases | Functions in: methyltransferase activity; Involved in: rRNA processing; Located in: chloroplast, cytoplasm |
| CL16405.Contig1 | CL11887.Contig1 | XP_012065465.1 0.0 | PREDICTED: cationic amino acid transporter 7, chloroplastic-like isoform X1 [Jatropha curcas] | AT3G10600.1 0 | Encodes a member of the cationic amino acid transporter (CAT) subfamily of amino acid polyamine choline transporters | Involved in: basic amino acid transport |
| CL16473.Contig2 | CL19711.Contig2 | AII80417.1 0.0 | RING-type E3 ubiquitin ligase [Vitis vinifera] | AT1G78420.2 1E-106 | RING/U-box superfamily protein | Functions in: zinc ion binding; Involved in: N-terminal protein myristoylation |
| CL15878.Contig1 | CL10269.Contig2 | XP_011096229.1 8e-86 | PREDICTED: E3 ubiquitin-protein ligase RNF170 [Sesamum indicum] | AT1G22510.1 2E-62 | RING/U-box protein with domain of unknown function (DUF 1232) | Located in: endoplasmic reticulum, integral component of membrane, mitochondrion, nucleus, plasma membrane, vacuolar membrane; Functions in zinc ion binding; has zinc ion binding |
| CL21626.Contig1 | CL12356.Contig4 | XP_011099836.1 5e-137 | PREDICTED: ribonuclease H2 subunit B [Sesamum indicum] | AT4G20325.1 4E-92 | Unknown protein | Located in: nucleus |
| CL7749.Contig1 | CL12436.Contig1 | XP_007218321.1 1e-163 | hypothetical protein PRUPE_ppa008412mg [Prunus persica] | AT5G16080.1 2E-41 | carboxyesterase 17 (CXE17) | Functions in: hydrolase activity; Involved in: metabolic process; Located in: cellular component unknown |
| CL8493.Contig2 | CL16524.Contig2 | XP_011027385.1 2e-113 | PREDICTED: psbP domain-containing protein 4, chloroplastic [Populus euphratica] | AT1G77090.1 9E-87 | Mog1/PsbP/DUF1795-like photosystem II reaction center PsbP family protein | Functions in: calcium ion binding; Involved in: photosynthesis; Located in: thylakoid, thylakoid lumen, chloroplast thylakoid lumen, chloroplast stroma, chloroplast |
| CL12773.Contig2 | CL19753.Contig3 | CBI29821.3 3e-116 | unnamed protein product [Vitis vinifera] | AT2G41670.1 4E-78 | Encodes SIN2 (SHORT INTEGUMENTS 2), a mitochondrial DAR GTPase. | Located in: chloroplast, mitochondrion; Functions in GTP binding |
| CL2395.Contig1 | CL16532.Contig1 | XP_011092401.1 0.0 | PREDICTED: uncharacterized protein LOC105172591 isoform X1 [Sesamum indicum] | AT5G64550.1 0 | loricrin-related; BEST Arabidopsis thaliana protein match is: loricrin-related (TAIR:AT5G09670.1) | Located in: nucleus; |
| CL13101.Contig2 | CL8027.Contig6 | AEC12208.1 9e-68 | JAZ1 [Maesa lanceolata] | AT1G19180.1 3E-34 | JAZ1 is a nuclear-localized protein Involved in: jasmonate signaling | Involved in: defense response to bacterium, flower development, jasmonic acid mediated signaling pathway, negative regulation of nucleic acid-templated transcription, pollen development, regulation of defense response, regulation of jasmonic acid mediated signaling pathway, response to jasmonic acid, response to wounding; Located in: nucleus; Functions in protein binding; has protein binding, transcription corepressor activity |
| Unigene25185 | CL13662.Contig1 | XP_006374872.1 2e-103 | Unknown protein | AT3G53010.1 1E-69 | Domain of unknown function (DUF303) | Located in: endomembrane system |
| Unigene26608 | CL13755.Contig1 | XP_014493313.1 1e-16 | TIMELESS-interacting protein | AT3G02820.1 6E-62 | zinc knuckle (CCHC-type) family protein | Functions in: zinc ion binding, nucleic acid binding; Involved in: cell cycle, replication fork protection, response to DNA damage stimulus; Located in: nucleus, chloroplast |
| CL16503.Contig1 | CL13816.Contig1 | XP_012087118.1 5e-32 | PREDICTED: non-specific lipid-transfer protein [Jatropha curcas] | AT1G62790.2 2e-19 | Bifunctional inhibitor/lipid-transfer protein/seed storage 2S albumin superfamily protein | Involved in: lipid transport; Located in: anchored component of membrane; Functions in lipid binding |
| CL4147.Contig1 | CL14050.Contig2 | XP_010275589.1 1e-159 | vestitone reductase | AT4G33360.1 E-121 | Encodes an NAD+-dependent dehydrogenase that oxidizes farnesol more efficiently than other prenyl alcohol substrates | Involved in: farnesol metabolic process, negative regulation of abscisic acid-activated signaling pathway, terpenoid metabolic process; Located in: endoplasmic reticulum, nucleus, plasma membrane, vacuolar membrane, vacuole; has catalytic activity, coenzyme binding, farnesol dehydrogenase activity |
| Unigene40961 | CL14083.Contig1 | XP_009600592.1 3e-171 | uncharacterized protein LOC104096018 isoform X2 | AT2G39580.1 2E-91 | CONTAINS InterPro DOMAIN/s: Putative zinc-finger domain (InterPro:IPR019607) | Located in: nucleus |
| CL1414.Contig3 | CL4570.Contig1 | XP_009620471.1 1e-111 | PREDICTED: uncharacterized protein LOC104112297 [Nicotiana tomentosiformis] | AT3G32930.1 2E-80 | Unknown protein | Located in: chloroplast envelope |
| CL14161.Contig3 | CL9547.Contig1 | XP_004307916.1 6e-174 | PREDICTED: peroxidase 5-like [Fragaria vesca subsp. vesca] | AT1G05260.1 1E-76 | Encodes a cold-inducible cationic peroxidase that is Involved in: the stress response | Involved in: hyperosmotic salinity response, plant-type cell wall organization, response to cold, response to desiccation; Located in: endoplasmic reticulum, extracellular region, plant-type cell wall; has heme binding, metal ion binding, peroxidase activity |
| CL16809.Contig1 | CL17259.Contig1 | XP_003632272.1 5e-90 | PREDICTED: shugoshin-1-like [Vitis vinifera] | AT3G10440 7e-18 | Encodes a protein that protects meiotic centromere cohesion | Involved in: maintenance of meiotic sister chromatid cohesion; Located in: chromosome, centromeric region, nucleus |
| CL6777.Contig3 | CL10436.Contig1 | BAB12439.1 2e-153 | gibberellin 3b-hydroxylase No3 [Lactuca sativa] | AT1G80340.1 2E-86 | Encodes a protein with gibberellin 3 β-hydroxylase activity | Involved in: gibberellin biosynthetic process, oxidation-reduction process, response to red light, response to red or far red light, seed germination; Located in: cytoplasm; has gibberellin 3-beta-dioxygenase activity, metal ion binding |
| CL16420.Contig1 | CL14584.Contig1 | CDP15822.1 3e-35 | unnamed protein product [Coffea canephora] | AT5G27330.1 3E-27 | Prefoldin chaperone subunit family protein | Located in: endoplasmic reticulum, integral component of membrane |
| CL17520.Contig1 | Unigene25976 | XP_009759851.1 0.0 | probable inactive leucine-rich repeat receptor-like protein kinase At5g20690 | AT3G42880.1 3E-151 | Leucine-rich repeat protein kinase family protein; | Functions in: protein serine/threonine kinase activity, kinase activity, ATP binding; Involved in: transmembrane receptor protein tyrosine kinase signaling pathway, protein amino acid phosphorylation; Located in: endomembrane system |
| CL10103.Contig1 | CL19627.Contig2 | XP_007199617.1 4e-62 | hypothetical protein PRUPE_ppa007168mg [Prunus persica] | AT4G38710.2 4E-55 | glycine-rich protein | Functions in: translation initiation factor activity; Involved in: translation, translational initiation; Located in: cytoplasm, nucleus |
| Unigene33895 | CL18954.Contig2 | CDP08716.1 0.0 | unnamed protein product [Coffea canephora] | AT5G41760.2 5E-154 | Nucleotide-sugar transporter family protein | Functions in: nucleotide-sugar transmembrane transporter activity, CMP-sialic acid transmembrane transporter activity; Involved in: carbohydrate transport, nucleotide-sugar transport; Located in: endomembrane system, integral to membrane, Golgi membrane |
| CL19344.Contig1 | CL22209.Contig1 | XP_009595321.1 0.0 | PREDICTED: phosphoenolpyruvate/phosphate translocator 1, chloroplastic-like [Nicotiana tomentosiformis] | AT5G33320.1 7E-150 | Encodes a plastid inner envelope protein PPT (phosphoenolpyruvate/phosphate translocator) that catalyzes the transport of phosphoenolpyruvate and phosphate across the inner envelope membrane of plastids | Involved in: carbohydrate transmembrane transport, phosphoenolpyruvate transmembrane transport, phosphoenolpyruvate transport, phosphoglycerate transport; Located in: chloroplast, chloroplast envelope, mitochondrion, plastid, plastid inner membrane; has antiporter activity, phosphoenolpyruvate: phosphate antiporter activity, phosphoglycerate transmembrane transporter activity |
| CL3018.Contig1 | CL19375.Contig1 | NP_001268170.1 2e-172 | Aux/IAA protein | AT5G65670.2 9E-95 | auxin (indole-3-acetic acid) induced gene The mRNA is cell-to-cell mobile | Involved in: auxin-activated signaling pathway, regulation of transcription, DNA-templated, response to auxin, transcription, DNA-templated; is downregulated by response to cyclopentenone; Located in: nucleus; has protein dimerization activity, transcription factor activity, sequence-specific DNA binding |
| CL1968.Contig3 | CL20698.Contig2 | XP_010644590.1 3e-53 | PREDICTED: uncharacterized protein LOC104877623 isoform X2 [Vitis vinifera] | AT1G55860.1 e-19 | encodes a ubiquitin-protein ligase containing a HECT domain. There are six other HECT-domain UPLs in Arabidopsis | Involved in: protein ubiquitination, protein ubiquitination Involved in: ubiquitin-dependent protein catabolic process, ubiquitin-dependent protein catabolic process; Located in: cytosol, membrane, mitochondrion, nucleus, plasmodesma, ubiquitin ligase complex; not Located in: cytosol; has ligase activity, ubiquitin-protein transferase activity |
| Unigene22066 | CL19852.Contig1 | XP_006365676.1 9e-81 | PREDICTED: transcription factor RF2a-like [Solanum tuberosum] | AT5G04840.1 3e-44 | bZIP protein | Functions in: DNA binding, sequence-specific DNA binding transcription factor activity; Involved in: regulation of transcription, DNA-dependent |
| CL20109.Contig1 | Unigene41728 | XP_010650542.1 9e-150 | PREDICTED: uncharacterized protein LOC104879453 isoform X2 [Vitis vinifera] | AT1G05950.1 2e-51 | Unknown protein | Located in: nucleus |
| CL202.Contig11 | Unigene30729 | XP_007039397.1 1e-89 | Uncharacterized protein TCM_015652 [Theobroma cacao] | AT4G24130.1 e-58 | Protein of unknown function, DUF538 | Located in: cytoplasm, nucleus |
| CL22102.Contig1 | CL1089.Contig1 | XP_007027469.1 9e-66 | Nucleic acid binding protein, putative [Theobroma cacao] | AT2G28200.1 6e-38 | C2H2-type zinc finger family protein | Functions in: sequence-specific DNA binding transcription factor activity, zinc ion binding, nucleic acid binding; Involved in: regulation of transcription; Located in: intracellular |
| CL10902.Contig2 | Unigene4665 | Q9SEC2.1 1e-131 | Full=Peptide methionine sulfoxide reductase; AltName: Full=Peptide-methionine (S)-S-oxide reductase; Short=Peptide Met(O) reductase; AltName: Full=Protein-methionine-S-oxide reductase | AT4G25130.1 7e-90 | Encodes a chloroplast-localized methionine sulfoxide reductase that is a member of the MSRA family. Involved in: protection of chloroplasts from oxidative stress | Involved in: cellular protein modification process, cellular response to oxidative stress, oxidation-reduction process, protein repair, response to cytokinin, response to light stimulus; Located in: chloroplast, chloroplast envelope, chloroplast stroma, mitochondrion; has peptide-methionine (S)-S-oxide reductase activity |
| CL20591.Contig2 | CL5402.Contig5 | XP_009619321.1 2e-53 | PREDICTED: cucumber peeling cupredoxin-like [Nicotiana tomentosiformis] | AT1G08500.1 2e-23 | early nodulin-like protein 18 (ENODL18) | Functions in: electron carrier activity, copper ion binding; Located in: anchored to membrane |
| CL20647.Contig2 | Unigene36752 | XP_002282518.2 4e-50 | PREDICTED: uncharacterized protein LOC100251153 [Vitis vinifera] | AT2G33250.1 5e-18 | Unknown protein | Located in: integral component of membrane, mitochondrion |
| CL21711.Contig1 | CL6977.Contig2 | CDP04003.1 3e-54 | unnamed protein product [Coffea canephora] | AT5G48335.1 3e-21 | Unknown protein | Located in: chloroplast |
| CL21764.Contig2 | CL7589.Contig2 | XP_007215836.1 4e-125 | hypothetical protein PRUPE_ppa010211mg [Prunus persica] | AT3G07800.1 e-85 | Encodes a thymidine kinase that salvages DNA precursors | Involved in: DNA biosynthetic process, nucleotide biosynthetic process, phosphorylation; Located in: cytoplasm; has ATP binding, identical protein binding, thymidine kinase activity |
| CL21796.Contig2 | Unigene45778 | CDP05786.1 2e-174 | unnamed protein product | AT1G50940.1 e-123 | Encodes the electron transfer flavoprotein ETF alpha, a putative subunit of the mitochondrial electron transfer flavoprotein complex (ETF beta is At5g43430.1) in Arabidopsis | Involved in: fatty acid beta-oxidation using acyl-CoA dehydrogenase, lipid homeostasis; Located in: mitochondrial matrix, mitochondrion; Functions in copper ion binding; has acyl-CoA dehydrogenase activity, electron carrier activity, fatty-acyl-CoA binding, flavin adenine dinucleotide binding, oxidoreductase activity, acting on the CH-CH group of donors, with a flavin as acceptor |
