# Supplementary material for: Genetic Adaptation of Giant Lobelias (Lobelia aberdarica and Lobelia telekii) to Different Altitudes in East African Mountains
Source: Front Plant Sci. 2016 Apr 12;7:488. doi: 10.3389/fpls.2016.00488 (PMC4828460; doi:10.3389/fpls.2016.00488)
Supplement: Supplementary file 3 [file Table_3.DOCX]

**Supplementary Table 3.** GO terms significantly over-represented among the positively selected genes (PSGs), negatively selected genes (NSGs) and strongly negatively selected genes (SNSGs). 'Ml' stands for meaningless if any of the expected counts are less than 5. And 'Na' stands for not available of the p-value of Pearson Chi-Square test. The GO terms, which are over-represented in the NSGs or SNSGs, are highlighted by blue colour; the GO terms, which are over-represented in PSGs or NSGs, are highlighted by yellow colour. The data for making this table are provided in Supplementary data.

| **Cellular Component:** | | | | |
| --- | --- | --- | --- | --- |
| **Number** of genes associated to the GO term of the three datasets | **Percentage** of the associated gene number to the total provided gene number | **P-value** between the gene number of every two datasets | GO number | Annotation |
| PSGs : NSGs : SNSGs | PSGs : NSGs : SNSGs | PSGs vs. NSGs : PSGs vs. SNSGs : NSGs vs. SNSGs |  |  |
| 0 : 42 : 18 | 0.0 : 1.9 : 4.1 | Ml : Ml : 0.004 | GO : 0005743 | mitochondrial inner membrane |
| 0 : 51 : 18 | 0.0 : 2.3 : 4.1 | Ml : Ml : 0.028 | GO : 0031966 | mitochondrial membrane |
| 0 : 52 : 19 | 0.0 : 2.3 : 4.3 | Ml : Ml : 0.017 | GO : 0005740 | mitochondrial envelope |
| 0 : 64 : 22 | 0.0 : 2.9 : 5.0 | Ml : Ml : 0.020 | GO : 0044429 | mitochondrial part |
| 1 : 27 : 11 | 1.3 : 1.2 : 2.5 | Ml : Ml : 0.036 | GO : 0044445 | cytosolic part |
| 1 : 36 : 17 | 1.3 : 1.6 : 3.9 | Ml : Ml : 0.002 | GO : 0048046 | apoplast |
| 1 : 38 : 16 | 1.3 : 1.7 : 3.7 | Ml : Ml : 0.008 | GO : 0030312 | external encapsulating structure |
| 1 : 38 : 16 | 1.3 : 1.7 : 3.7 | Ml : Ml : 0.008 | GO : 0005618 | cell wall |
| 1 : 50 : 19 | 1.3 : 2.3 : 4.3 | Ml : Ml : 0.012 | GO : 0005840 | ribosome |
| 1 : 56 : 19 | 1.3 : 2.5 : 4.3 | Ml : Ml : 0.035 | GO : 0019866 | organelle inner membrane |
| 1 : 81 : 25 | 1.3 : 3.6 : 5.7 | Ml : Ml : 0.043 | GO : 0034357 | photosynthetic membrane |
| 16 : 556 : 138 | 20.8 : 25.0 : 31.6 | 0.397 : 0.056 : 0.004 | GO : 0044446 | intracellular organelle part |
| 16 : 556 : 138 | 20.8 : 25.0 : 31.6 | 0.397 : 0.056 : 0.004 | GO : 0044422 | organelle part |
| 2 : 107 : 32 | 2.6 : 4.8 : 7.3 | Ml : 0.124 : 0.031 | GO : 0005794 | Golgi apparatus |
| 2 : 71 : 25 | 2.6 : 3.2 : 5.7 | Ml : Ml : 0.010 | GO : 0030529 | ribonucleoprotein complex |
| 26 : 1081 : 248 | 33.8 : 48.6 : 56.8 | 0.010 : 0.000 : 0.002 | GO : 0044444 | cytoplasmic part |
| 3 : 173 : 63 | 3.9 : 7.8 : 14.4 | 0.207 : 0.011 : 0.000 | GO : 0043234 | protein complex |
| 3 : 245 : 51 | 3.9 : 11.0 : 11.7 | 0.047 : 0.040 : 0.695 | GO : 0016021 | integral to membrane |
| 35 : 806 : 131 | 45.5 : 36.3 : 30.0 | 0.100 : 0.007 : 0.012 | GO : 0005634 | nucleus |
| 37 : 1321 : 295 | 48.1 : 59.5 : 67.5 | 0.046 : 0.001 : 0.002 | GO : 0005737 | cytoplasm |
| 5 : 241 : 86 | 6.5 : 10.8 : 19.7 | 0.224 : 0.005 : 0.000 | GO : 0032991 | macromolecular complex |
| 6 : 278 : 70 | 7.8 : 12.5 : 16.0 | 0.216 : 0.061 : 0.047 | GO : 0031090 | organelle membrane |
| **Biological Process:** | | | | |
| **Number** of genes associated to the GO term of the three datasets | **Percentage** of the associated gene number to the total provided gene number | **P-value** between the gene number of every two input dataset | GO number | Annotation |
| PSGs : NSGs : SNSGs | PSGs : NSGs : SNSGs | PSGs vs. NSGs : PSGs vs. SNSGs : NSGs vs. SNSGs |  |  |
| 0 : 33 : 16 | 0.0 : 1.5 : 3.7 | Ml : Ml : 0.002 | GO : 0046165 | alcohol biosynthetic process |
| 1 : 75 : 28 | 1.3 : 3.4 : 6.4 | Ml : Ml : 0.003 | GO : 0046164 | alcohol catabolic process |
| 3 : 158 : 47 | 3.9 : 7.1 : 10.8 | 0.277 : 0.061 : 0.009 | GO : 0006066 | alcohol metabolic process |
| 2 : 139 : 50 | 2.6 : 6.3 : 11.4 | Ml : 0.018 : 0.000 | GO : 0010926 | anatomical structure formation |
| 2 : 92 : 28 | 2.6 : 4.1 : 6.4 | Ml : Ml : 0.037 | GO : 0016052 | carbohydrate catabolic process |
| 0 : 36 : 1 | 0.0 : 1.6 : 0.2 | Ml : Ml : 0.023 | GO : 0016117 | carotenoid biosynthetic process |
| 1 : 111 : 44 | 1.3 : 5.0 : 10.1 | Ml : 0.012 : 0.000 | GO : 0022607 | cellular component assembly |
| 3 : 182 : 61 | 3.9 : 8.2 : 14.0 | 0.173 : 0.014 : 0.000 | GO : 0044085 | cellular component biogenesis |
| 0 : 91 : 40 | 0.0 : 4.1 : 9.2 | Ml : 0.006 : 0.000 | GO : 0034622 | cellular macromolecular complex assembly |
| 0 : 98 : 40 | 0.0 : 4.4 : 9.2 | Ml : 0.006 : 0.000 | GO : 0034621 | cellular macromolecular complex subunit organization |
| 1 : 34 : 22 | 1.3 : 1.5 : 5.0 | Ml : Ml : 0.000 | GO : 0043094 | cellular metabolic compound salvage |
| 0 : 80 : 32 | 0.0 : 3.6 : 7.3 | Ml : Ml : 0.000 | GO : 0043623 | cellular protein complex assembly |
| 13 : 243 : 40 | 16.9 : 10.9 : 9.2 | 0.103 : 0.040 : 0.269 | GO : 0051716 | cellular response to stimulus |
| 4 : 107 : 11 | 5.2 : 4.8 : 2.5 | Ml : Ml : 0.033 | GO : 0051276 | chromosome organization |
| 0 : 55 : 19 | 0.0 : 2.5 : 4.3 | Ml : Ml : 0.030 | GO : 0009790 | embryonic development |
| 0 : 54 : 19 | 0.0 : 2.4 : 4.3 | Ml : Ml : 0.025 | GO : 0009793 | embryonic development ending in seed dormancy |
| 1 : 66 : 21 | 1.3 : 3.0 : 4.8 | Ml : Ml : 0.049 | GO : 0010154 | fruit development |
| 1 : 39 : 2 | 1.3 : 1.8 : 0.5 | Ml : Ml : 0.044 | GO : 0031047 | gene silencing by RNA |
| 0 : 92 : 34 | 0.0 : 4.1 : 7.8 | Ml : 0.011 : 0.001 | GO : 0006091 | generation of precursor metabolites and energy |
| 0 : 25 : 13 | 0.0 : 1.1 : 3.0 | Ml : Ml : 0.003 | GO : 0006096 | glycolysis |
| 2 : 86 : 31 | 2.6 : 3.9 : 7.1 | Ml : Ml : 0.003 | GO : 0019318 | hexose metabolic process |
| 0 : 94 : 40 | 0.0 : 4.2 : 9.2 | Ml : 0.006 : 0.000 | GO : 0065003 | macromolecular complex assembly |
| 0 : 101 : 40 | 0.0 : 4.5 : 9.2 | Ml : 0.006 : 0.000 | GO : 0043933 | macromolecular complex subunit organization |
| 1 : 26 : 11 | 1.3 : 1.2 : 2.5 | Ml : Ml : 0.028 | GO : 0006839 | mitochondrial transport |
| 1 : 74 : 28 | 1.3 : 3.3 : 6.4 | Ml : Ml : 0.002 | GO : 0046365 | monosaccharide catabolic process |
| 2 : 127 : 39 | 2.6 : 5.7 : 8.9 | Ml : 0.059 : 0.011 | GO : 0005996 | monosaccharide metabolic process |
| 1 : 33 : 17 | 1.3 : 1.5 : 3.9 | Ml : Ml : 0.001 | GO : 0009853 | photorespiration |
| 1 : 57 : 24 | 1.3 : 2.6 : 5.5 | Ml : Ml : 0.001 | GO : 0015979 | photosynthesis |
| 0 : 48 : 19 | 0.0 : 2.2 : 4.3 | Ml : Ml : 0.008 | GO : 0019684 | photosynthesis, light reaction |
| 12 : 321 : 43 | 15.6 : 14.4 : 9.8 | 0.780 : 0.133 : 0.010 | GO : 0046148 | pigment biosynthetic process |
| 12 : 275 : 39 | 15.6 : 12.4 : 8.9 | 0.402 : 0.071 : 0.041 | GO : 0051191 | prosthetic group biosynthetic process |
| 0 : 83 : 33 | 0.0 : 3.7 : 7.6 | Ml : Ml : 0.000 | GO : 0006461 | protein complex assembly |
| 0 : 83 : 33 | 0.0 : 3.7 : 7.6 | Ml : Ml : 0.000 | GO : 0070271 | protein complex biogenesis |
| 12 : 260 : 36 | 15.6 : 11.7 : 8.2 | 0.300 : 0.041 : 0.035 | GO : 0019507 | pyridine metabolic process |
| 12 : 275 : 39 | 15.6 : 12.4 : 8.9 | 0.402 : 0.071 : 0.041 | GO : 0009889 | regulation of biosynthetic process |
| 12 : 275 : 39 | 15.6 : 12.4 : 8.9 | 0.402 : 0.071 : 0.041 | GO : 0031326 | regulation of cellular biosynthetic process |
| 0 : 33 : 14 | 0.0 : 1.5 : 3.2 | Ml : Ml : 0.013 | GO : 0010817 | regulation of hormone levels |
| 12 : 260 : 36 | 15.6 : 11.7 : 8.2 | 0.300 : 0.041 : 0.035 | GO : 0051171 | regulation of nitrogen compound metabolic process |
| 12 : 258 : 35 | 15.6 : 11.6 : 8.0 | 0.287 : 0.033 : 0.028 | GO : 0019219 | regulation of nucleobase, nucleoside, nucleotide and nucleic acid metabolic process |
| 10 : 243 : 33 | 13.0 : 10.9 : 7.6 | 0.572 : 0.112 : 0.034 | GO : 0051252 | regulation of RNA metabolic process |
| 0 : 34 : 13 | 0.0 : 1.5 : 3.0 | Ml : Ml : 0.036 | GO : 0051788 | response to misfolded protein |
| 2 : 76 : 27 | 2.6 : 3.4 : 6.2 | Ml : Ml : 0.006 | GO : 0022613 | ribonucleoprotein complex biogenesis |
| 2 : 72 : 24 | 2.6 : 3.2 : 5.5 | Ml : Ml : 0.021 | GO : 0042254 | ribosome biogenesis |
| 1 : 64 : 21 | 1.3 : 2.9 : 4.8 | Ml : Ml : 0.036 | GO : 0048316 | seed development |
| 0 : 37 : 1 | 0.0 : 1.7 : 0.2 | Ml : Ml : 0.021 | GO : 0016108 | tetraterpenoid metabolic process |
| 10 : 269 : 37 | 13.0 : 12.1 : 8.5 | 0.816 : 0.204 : 0.029 | GO : 0006350 | transcription |
| 4 : 66 : 27 | 5.2 : 3.0 : 6.2 | Ml : Ml : 0.001 | GO : 0006412 | translation |
| **Molecular Function:** | | | | |
| **Number** of genes associated to the GO term of the three datasets | **Percentage** of the associated gene number to the total provided gene number | **P-value** between the gene number of every two input dataset | GO number | Annotation |
| PSGs : NSGs : SNSGs | PSGs : NSGs : SNSGs | PSGs vs. NSGs : PSGs vs. SNSGs : NSGs vs. SNSGs |  |  |
| 0 : 45 : 16 | 0.0 : 2.0 : 3.7 | Ml : Ml : 0.037 | GO : 0008233 | peptidase activity |
| 1 : 29 : 13 | 1.3 : 1.3 : 3.0 | Ml : Ml : 0.010 | GO : 0016853 | isomerase activity |
| 1 : 39 : 17 | 1.3 : 1.8 : 3.9 | Ml : Ml : 0.004 | GO : 0003735 | structural constituent of ribosome |
| 1 : 52 : 21 | 1.3 : 2.3 : 4.8 | Ml : Ml : 0.004 | GO : 0005198 | structural molecule activity |
| 19 : 774 : 161 | 24.7 : 34.8 : 36.8 | 0.065 : 0.039 : 0.421 | GO : 0003824 | catalytic activity |
| 3 : 258 : 57 | 3.9 : 11.6 : 13.0 | 0.036 : 0.021 : 0.397 | GO : 0016787 | hydrolase activity |
| 7 : 129 : 15 | 9.1 : 5.8 : 3.4 | Ml : Ml : 0.045 | GO : 0030528 | transcription regulator activity |
