# Supplementary figures and images for: Genetic Adaptation of Giant Lobelias (Lobelia aberdarica and Lobelia telekii) to Different Altitudes in East African Mountains
Source: Front Plant Sci. 2016 Apr 12;7:488. doi: 10.3389/fpls.2016.00488 (PMC4828460; doi:10.3389/fpls.2016.00488)

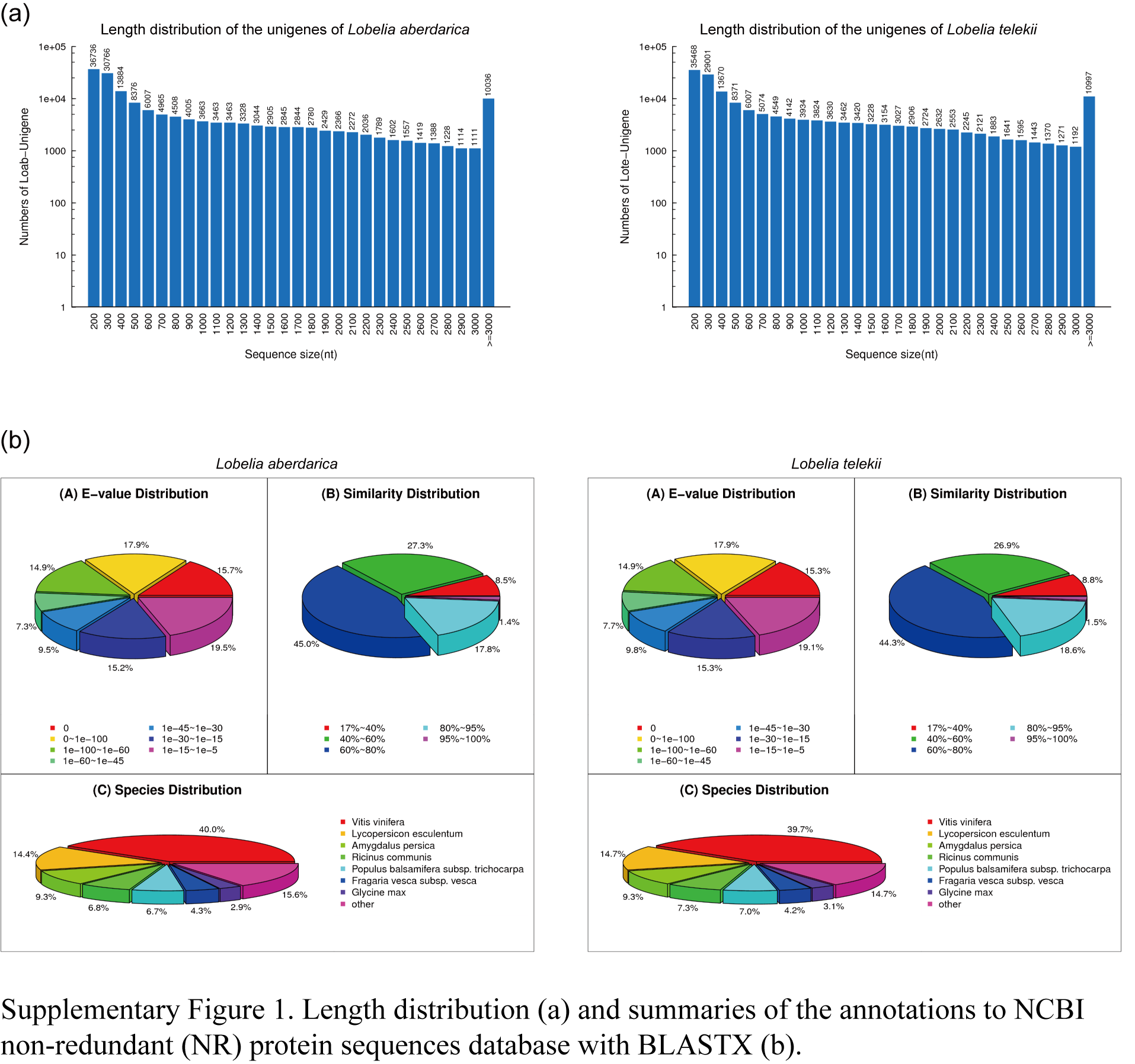

Supplement: Supplementary file 5 [file Image_1.TIF]
